# The contributions of language and inhibitory control to false belief reasoning over time

**DOI:** 10.3389/fpsyg.2024.1455941

**Published:** 2025-01-03

**Authors:** Jill G. de Villiers, Peter de Villiers

**Affiliations:** Department of Psychology, Smith College, Northampton, MA, United States

**Keywords:** theory-of-mind, complements, structural equation, language, inhibitory control, longitudinal

## Abstract

**Introduction:**

The role of language in false belief reasoning has been much debated for twenty-five years or more, especially the relative contributions of general language development, complement syntax, vocabulary, and executive function. However, the empirical studies so far have fallen short, in that they generally have too few participants for adequate statistical modeling; they do not include control variables; or they are cross-sectional rather than longitudinal, making inferences about causal direction much more tenuous.

**Methods:**

The present study considers the role of these different variables in the development of false belief reasoning over several months of testing, with 258 children aged three to five years. The children are also from under-resourced communities, broadening the populations that generally contribute such data.

**Results:**

A cross-sectional and a longitudinal regression analysis reveals the contribution of each variable to the children’s success on the false belief measures. Finally, a structural equation model tests the relative contribution of the different potential factors over time, how they interact, and change. The model is an excellent fit to the data. Inhibitory control, complement comprehension and vocabulary all have effects on false belief reasoning at the first time point (T1). However, at T3, the major proximal contribution is the child’s comprehension of complements, though the longitudinal pathways of vocabulary and inhibitory control also pave the way.

**Discussion:**

Our data confirm the specific contribution of complement syntax but also makes clear, as do training studies, that a certain amount of preparedness in vocabulary and in executive function skills is also necessary.

## 1 Introduction

### 1.1 The course of early theory of mind

Theory of mind, defined as the understanding that others’ mental states may be different from one’s own, has been studied exhaustively for four decades now, and there remain many debates about the influences on the developmental path children take. The general developmental course itself is not disputed, though the extent of cross-cultural variation is still not established. The stepwise development defined by Wellman and Liu (2004) in terms of definable and well-matched experimental tasks is generally accepted, though there is still uncertainty about the causal necessity of each stage. The first step in their developmental sequence, at around age 18 months, is understanding others’ desires, that is, recognizing that two people may want different things than the child does (Repacholi and Gopnik, 1997). Following this, the child comes to understand that people will act according to their beliefs, even if different from the child’s. Understanding that “seeing leads to knowing,” or knowledge access, is slightly later. Realizing that others can have false beliefs, when the child has evidence to the contrary, comes in around age four or five years. Finally, understanding “hidden emotions” is found to be a still later development (Wellman et al., 2011). However, the sequence of middle steps may be different for Chinese children, where understanding different beliefs may follow knowledge access (Wellman et al., 2006; Yu and Wellman, 2024). Still further theory of mind skills, such as second order false belief reasoning (Miller, 2009) and understanding sarcasm and irony (Filippova and Astington, 2010; Winner, 1997) do not develop until early school age.

Digging deeper, it is clear there are also important developments before age two. In the first year of life, children are sensitive to others’ intentions and goals, and differentiate between intentional and unintentional actions (Woodward, 2009). When learning their first language, children make use of shared attention with a caregiver to establish the referents of early words (Baldwin, 1995; Tomasello, 1999). Furthermore, infants in the second year of life can track which objects another person has seen before, and will show that person toys that they have not seen, or point out to them where an object was hidden (Tomasello, 1999).

Why has false belief reasoning occupied center stage in the literature about theory of mind? Philosophers and psychologists have considered the achievement of false belief understanding to be a watershed not only in early child development (Dennett, 1978; Wellman et al., 2001) but also in assessing the theory of mind capabilities of other species (Call and Tomasello, 2008; Premack and Woodruff, 1978) and recently, of A.I. (Strachan et al., 2024) In a false-belief task, you must hold in memory alternative perspectives within a given context (e.g., where a target object actually is and where a character thinks it is). You then must also inhibit the prepotent response of selecting a perspective that is congruent with your own (i.e., selecting where you know the target object to be) (Leslie et al., 2005). To understand another’s false beliefs is to entertain ideas inconsistent with the reality you know, and to reason from that representation about either the cause or the consequence of that belief. Westra and Nagel (2021) refer to this as *non-factive* reasoning, in contrast to the *factive* reasoning that is entailed in recognizing what someone knows. In recognizing knowledge, you pay attention to whether the person has perceptual access to the thing or event in question, and since you share the perception, there is no contradiction in perspective. We know that quite young children are active observers of attention and perceptual access, but do not seem attentive to mistaken beliefs.

Since the work of Wellman and Liu (2004), there has been an explosion of research on infant theory of mind, with some researchers claiming that given the right tasks, infants can be shown to recognize that another character has a false belief (Baillargeon et al., 2010; Scott and Baillargeon, 2017). The theory of mind tasks used with infants are called implicit tasks, because no behavioral decision is required from the infant, that is, there is no step of response selection. The first type of task uses the length of gaze as the measure. Infants in the second year of life or even younger have been shown to gaze for a more protracted time at events in which a human character acts in a way contrary to expectation, namely, a way that is not in keeping with the belief they should have formed (Onishi and Baillargeon, 2005). For example, in a version of an unseen displacement task, they are surprised when the character being observed goes to a location where an object really is, when the character did not see it move there. In a second task type, infants look expectantly at a location where a character should go, based on where that character falsely believes something to be (Southgate et al., 2007). In a third design, very young children come to the assistance of another individual specifically if that person was not witness to how something works (Buttelmann et al., 2009). Clearly, success at a young age on implicit tasks calls into question the necessity of language as an aid to reasoning.

The age gap between these tasks and explicit false belief mastery around age four requires explanation. On the account provided by several researchers (e.g., Baillargeon et al., 2010), the gap is a result of the task demands of explicit tasks, namely, response selection sets the bar too high for infants. On this account infants have implicit understanding of others’ beliefs, as rich as that of older children or adults (Baillargeon et al., 2018). However, others ask: Are the infants in these studies acting on the basis of a belief attribution, or something simpler (Southgate, 2013)? The most reductive explanation is that the infant is responding to some accidental but correlated feature of the set-up (Heyes, 2014). Some have found a lack of connection between the looking tasks and the helping tasks (e.g., Poulin-Dubois and Yott, 2018), arguing against abstract mental states underlying both. Other theorists suggest that the child is responsive to a behavioral rule, such as “people go to where they last saw something” (Perner and Ruffman, 2005). Southgate and Vernetti (2014) suggested that infants may be able to follow an agent’s point of view at ages as young as 6 months, but the difference is that they do not yet contrast it with their own. In a widely-cited account, Apperly and Butterfill (2009) proposed that infants may “register” another’s belief rather than represent it. Registration allows infants to trace and track another’s belief but in a limited way that betrays it as different from mature representation, for example, if the belief being tracked has relationships to other mental states, or involves subtle properties not easily captured by vision. As one example, registration may be restricted to tracking expected location rather than how a person construes an object (see also Low, 2010). On Apperly and Butterfill’s two-systems account, the registration that infants use gives way to real representation in older children.

Unfortunately, these fascinating ideas remain in doubt due to a replicability crisis. Many failures to replicate the findings have been reported across developmental labs, and the difficulty is that failures do not get published (Kulke and Rakoczy, 2018; Kulke et al., 2018; Rakoczy, 2012; but see Baillargeon et al., 2018). Brooks and Meltzoff (2015) found continuity in a small group of nineteen children from the first implicit understanding in anticipatory looking in infancy at 10.5 months through to explicit false belief development at age 4.5 years. However, implicit theory of mind tasks used beyond infancy do not show a consistent relationship to explicit tasks with the same preschool children (Low, 2010; Grosse Wiesmann et al., 2017). Nevertheless, the putative gap between infant and four-year-old theory of mind needs explanation and a popular suggestion is that the child needs to develop a mature executive function before success on explicit false belief tasks, because of the cognitive demands of the task. We will refer to this as the *executive function hypothesis*.

### 1.2 Executive functions and theory of mind

Executive function development is on a time course that has some correspondence with theory of mind changes, with some significant maturation happening around four or five years of age. Two aspects in particular have been highlighted as potential determinants of the change in false belief reasoning development in previous research. One is *working memory* (Brandt et al., 2023; Carlson et al., 2002), and the second is *inhibitory control* (Carlson et al., 2015; Carlson and Moses, 2001; Carlson et al., 2002). In an explicit false-belief task, it is necessary to hold alternatives in memory (e.g., what an object actually is and what someone thinks it is). The child also has to inhibit the alternative that is in keeping with their own beliefs, and to resist that lure of reality. Inhibitory control would also seem to be prerequisite for the step of inhibiting the reality response. In fact, in an important meta-analysis of previous work on executive function and false belief reasoning, Devine and Hughes (2014) report that inhibitory control is a reliable predictor of false belief understanding in preschoolers. Nevertheless, is it important to consider the role of executive function in conjunction with the role of another significant skill developing over the preschool years, namely language.

### 1.3 Language and theory of mind

Alternative proposals have highlighted the role of language in the process of theory of mind development (de Villiers and de Villiers, 2009), and some theorists argue that language is an alternative to bridge the gap from infant implicit understanding to explicit false belief reasoning (Apperly and Butterfill, 2009). Studies have found that the child’s own language appears to be a key factor in mastery of explicit false belief understanding in young children (Astington and Baird, 2005; San Juan and Astington, 2012). However, is it learning mental state vocabulary (Shatz, 1994), engaging in rich discourse (Harris et al., 2005; Nelson, 2005), or acquiring grammatical structures that contribute to general reasoning (Farrar et al., 2017; Ruffman et al., 2002). In particular, are specific syntactic achievements necessary (de Villiers and de Villiers, 2000, 2014)?

#### 1.3.1 Sentential complements

Explicit false belief reasoning requires minimally two processes:

- The representation of alternatives, one of which is false.
- The ability to inhibit a prepotent response in response selection.

The representation of false alternatives is much more easily done with language than with images or even words (Nordmeyer and de Villiers, 2019). Neither images nor words can be false, as they are not propositions in themselves. Of course, one can form different images representing different perspectives, but not one that is false, or negative, without something extra.

“Consider, for example, negation. It’s easy to tell somebody that it’s not going to rain. Try drawing them a picture of it’s not going to rain … Think about trying to draw a picture of “there’s not a giraffe standing beside me” (Fodor, 1994).

This is trivially easy to do, unless one needs to recognize what it is a picture of. Language itself has rich ways of representing not only different perspectives, for example through deixis (de Villiers, 2018), but it can also represent negation and other logical terms that images do not. The unique part about certain embedded sentences, or sentential complements, is that they can represent a false proposition inside a true sentence.

de Villiers (2007) has argued that complements constitute an example of language-as-cognitive tool that has special utility in representing the states of others’ minds. The complement in (1) is distinct from the adjunct clause in (2), because the embedded proposition in (1) can be false:

1. Miriam said *that she baked the bread*.
2. Miriam relaxed *after she baked the bread*.

The complement structure only occurs under communication and mental state verbs. Thus, complements can express mistakes and lies, and with mental state verbs like *think* or *believe,* they can describe false beliefs. Possible worlds can be described in which those propositions could be true, namely, worlds in the mind of the sentence subject. Philosophers call these *propositional attitudes*, in which sentences express false propositions as belonging to another’s mind or perspective. While it is true that specific vocabulary words exist to name the state of a false belief, on its own a verb such as “deluded” (3) does not capture the content of the delusion, and the contents of propositional attitudes (4) matter in predicting what the subject will do, or in explaining what they have done.

1. Miriam’s friend was deluded
2. Miriam’s friend thought that Miriam baked the bread.

The properties that sentential complements have for capturing mental states are discussed extensively in the literature on propositional attitudes (Davidson, 1984; Richard, 1990; Segal, 1998).

The special characteristic of complements is their contrast of two perspectives, making them uniquely suited to the representation of propositional attitudes such as belief. There are multiple more subtle differences among complements in English and other languages, representing a network of precise decisions in the learning process for children to master (de Villiers and Roeper, 2016). One difference is whether the embedded clause is tensed: non-finite clauses have neither tense, nor an independent truth-value (5):

1. Anna wanted him to play in the garden.

The phrase “him to play in the garden” is not something one can evaluate for truth, unlike the final clause in a tensed complement (6):

1. Anna thought he was playing in the garden.

Children master nonfinite complements (7) before they master tensed, finite complements (8), and do not make “reality” mistakes with the former, even when there is a contrast in what happened (de Villiers et al., 2012),

1. Anna told him to play in the garden, but he went to sleep instead.

What did Anna tell him to do? – Three-year-olds: play in the garden.

1. Anna said he was playing in the garden, but he went to sleep instead.

What did Anna say he was doing? – Three-year-olds: sleeping.

De Villiers (1995) argued that much of this grammar is established with verbs of communication, as they share many of the same linguistic distinctions with mental verbs, but the difference is that communication events are overt and not covert as with hidden mental states. The child can understand the reference of a verb like “say,” and has undoubtedly heard household arguments about events of speech in which truth can be checked:

1. You said you fed the dog but you did not!

Think of the mindreading needed instead to produce:

1. You thought you fed the dog but you did not!

At the end of this acquisition process, the child understands the syntax of finite, non-factive, complements. However, the fine-grained distinctions in meaning among mental verbs-which number in the hundreds-still take time and experience in the world, a process beyond syntax itself, and into discourse pragmatics.

But how could acquiring the structure of complements play a role in establishing the very concept of false belief? It should surely be otherwise, namely that first the child understands false beliefs and second, learns to encode them in complements. Then one would see a correlation between the two achievements, but the lines of causation would be the other way round. Further, many researchers argue in favor of a bidirectional relationship between ToM and language. On the one hand, the child’s growing understanding of people’s actions, goals, and desires surely provides some conceptual grounding for the meaning involved. Mental states are unobservable entities, so hearing a new word like “think” or “forget” will prompt children to consider what these terms might be labeling, and to conceptualize the distinctions among them (Pyers and Senghas, 2009; Low, 2010). Importantly though, the appearance of these verbs in sentence frames with complements sets limits on their possible meaning (Papafragou, 2001). Could sentential complement structures like (1) have a particular enabling function for false belief reasoning? This particular debate has resulted in much attention, both theoretically (Hinzen, 2007; de Villiers, 2007) and empirically (Boeg Thomsen et al., 2021; Brandt et al., 2023; Farrar et al., 2017; Cheung et al., 2004).

#### 1.3.2 Empirical studies

We know that children begin using verbs such as *think* and *know* from an early age (Bartsch and Wellman, 1995; Diessel and Tomasello, 2001; Shatz, 1994; Shatz et al., 1983) but their first uses may be less like expressions of propositional attitudes than like stereotyped forms, often self-referent, with narrow functions. The forms in (11) and (12) do not capture the contrast between truth and reality, or contrasts across minds, unlike the form in (1).

1. I do not know (used as an escape from questioning).
2. I think it’s a dog (I think used as “maybe”).

Examples like (12) led Diessel and Tomasello (2001) to argue that children treat the high-frequency string “I think” more like an adverb, without explicit mental-state reference. As a result, hearing examples like (12) with first-person subjects might not help children to recognize false-belief understanding. The very first expressions of third person propositional attitudes seem to emerge around 3 or 3.5 years in spontaneous speech, and occur more rarely (Bartsch and Wellman, 1995). However, in experimental settings when children are tested about understanding the forms, consistent difficulty is revealed. For instance, de Villiers (1999) arranged scenarios as in (13) in which characters made statements that were either lies or mistakes, such as:

1. The woman said she found her slipper. But look, it was really a mouse.

What did the woman say she found?

Three-year-olds consistently answer “mouse,” even though the answer is provided in the sentence and one can argue that no “mind reading” is necessary in the situation. Answering correctly entails understanding the discourse and reconstructing what the question refers to, which is only possible if the syntax of complements is mastered. Four-and five-year-olds answer “slipper,” as do adults. A longitudinal study of three-and four-year-olds by de Villiers and Pyers (2002) and a very large cross-sectional study of 1,000 children aged four to ten in the standardization of the DELV language assessment test (Seymour et al., 2003) exposed the time course and uniformity of this development (de Villiers et al., 2003).

The finding of correlation between complements and performance on explicit false belief reasoning tasks has been documented now in several different languages: English (e.g., de Villiers and Pyers, 2002; Brandt et al., 2023; Boeg Thomsen et al., 2021) German (Perner et al., 2003; Grosse Wiesmann et al., 2017); Danish (Knuppel et al., 2007), Mandarin (Mo et al., 2014; Guo et al., 2022; Brandt et al., 2023; Li and Leung, 2024), and ASL (Schick et al., 2007). Most but not all of the studies exploring the connection have used the comprehension test originating in the work of de Villiers (1995). In a variant, Brandt et al. (2016) evaluated children’s comprehension of complements using first versus third person subjects, and found that only performance with third-person complements correlated with 4-year-olds’ false belief performance. Aksu-Koç et al. (2005) found that production of complements in Turkish predicted false belief reasoning better than the production of evidential markers did. In Dutch, de Mulder et al. avoided the truth contrasts of the complement comprehension task and asked the children simply to report what different people reported happening, when the truth of the matter was unknown. Success on that task proved unrelated to false belief reasoning. Yet in Brandt et al., in English, truth contrasts were also side-stepped: the child had to recall what was said in a complement across an intervening sentence that was merely a distractor, but did not deny its truth. In contrast to the results in de Mulder et al., performance on this task was well correlated with false beliefs. In the work in German by Grosse Wiesmann et al. (2017), the child’s imitation of complements was used instead, because imitation can betray whether children understand the crucial difference in placement of the complementizer in German. The children’s skill at accurate imitation of the complements was connected to their false belief reasoning. Hence, the basic result seems to survive most variations in the task used (but see Brandt et al. (2023) on complement production).

The effects are less clear in children learning Cantonese (Cheung et al., 2004; Cheung et al., 2009; Tardif et al., 2007), a language in which the surface markers of complementation are virtually non-existent and there is no wh-question movement. Tardif et al. (2007) reported a large longitudinal study of children learning Cantonese in Hong Kong, and though she found significant correlations between complement comprehension on the de Villiers and Pyers (2002) “memory for complements” task and false belief understanding, the children overall were surprisingly poor at the complement comprehension test, even at age six. The complements did not seem to be prerequisite for false belief reasoning in Cantonese, a finding echoed in a recent longitudinal path analysis by Siu and Cheung (2022). Yet in closely-related Mandarin, Guo et al. (2022) present robust evidence of a correlation between complements and false belief reasoning in both typical children and children with autism [see also Li and Leung, 2024 (this issue)].

Brandt et al. (2023) explored the CHILDES data on parent–child conversations in Mandarin and found that the parents, unlike their English counterparts, do not show the frequent use of “I think” as an epistemic adverb. In their experimental study, Brandt et al. (2023) found that discriminating the use of the non-factive verb *falsely think* from *know* complements was related to false belief understanding, regardless of the subject being first or third person. English speaking children, however, were affected by the person of the verb in a parallel study. Following a suggestion by Tardif and Wellman (2000) on the relative lack of discourse on mental states from Chinese parents to children, Mo et al. (2014) trained children on complements using verbs of communication. They found the training paid off in children’s improvement on false belief reasoning.

When children are language delayed, either by DLD (Developmental Language Disorder), by autism spectrum disorders, or by deafness, the path of development can be stretched out in a way that allows researchers to see the order of mastery (Durrleman et al. 2016). The case of language-delayed deaf children is especially relevant here, as these children do not usually have other associated developmental problems or neurodivergent minds, but may just have restricted access to the primary input. Children who are born to hearing parents and do not have the benefit of exposure to signed languages often struggle to learn oral language, and are delayed several years as a result. Schick et al. (2007) and Tager-Flusberg and Joseph (2005) studied these children’s language and false belief development, and report delays in false belief understanding concomitant with their language delay. Both vocabulary and complement comprehension predicted their false belief understanding, even though the false belief tasks were designed to be as minimally verbal as possible. That is, the language required for the task was ruled out as a variable. Since the linguistic skill is needed equally for non-or low-verbal false belief tasks, it suggests that language is needed to develop the false belief reasoning, not just to understand the classic tasks. In an extreme case in Nicaragua, researchers for several decades have studied the invention of a sign language NSL (Nicaraguan Sign Language) by deaf children sent away to special boarding schools. The first generation of such signers had relatively incomplete grammars, without sophisticated grammatical ways to indicate other people’s perspectives. The deficiency was then remedied in successive generations of learners. The developmental inversion is that older learners of NSL can be less competent than younger learners. Pyers (2004) and Pyers and Senghas (2009) found that those signers who could express propositional contents under mental state verbs were able to do false belief reasoning (in a nonverbal test), but those signers who did not, failed them even as adults. Incomplete language development hinders theory of mind development even in adult populations.

#### 1.3.3 Explanatory accounts

Despite the empirical data primarily in support of complements as a factor in false belief development, the explanation is still very much debated. Sentential complements represent one example of complex language, possibly the apex of syntactic success in the preschool years. Perhaps general language development (usually indexed by vocabulary) or general syntactic skill are the real predictors of false belief, and complements are just representative of that stage of acquisition (Ruffman, et al., 2002). Call this the *general language* hypothesis. Some have argued that complements reflect the kind of rich discourse that encourages false belief reasoning, that is, the content of the theory about minds is exposed by such conversation (Harris et al., 2005; Nelson, 2005; Tompkins et al., 2018). This can be called the *discourse content* hypothesis. Alternatively, developing complement structures might scaffold the kind of careful reasoning that the scenarios entail, allowing the child to entertain multiple perspectives on the world and successfully choose among them (for a rich discussion of these and other alternatives, see San Juan and Astington, 2017; de Villiers, 2021). This we call the *complements facilitate reasoning* hypothesis.

Empirical results that might allow differentiation of these alternatives are ambivalent, as not all the studies included both general language and complementation measures in the same investigation. For example, Low (2010) found that understanding sentential complements predicted standard false belief tasks in a cross-sectional sample of English-speaking children, once age, nonverbal ability and implicit false belief scores were controlled, but no alternative indices of language were included. Meta-analyses provide some help with this question, but the results are somewhat mixed. In a meta-analysis in Milligan et al. (2007) considered all the language measures used by studies to that date. Various different language measures, including syntax (29%), general language (27%), semantics (23%), and receptive vocabulary (12%) made significant contributions to false belief understanding. However, memory for complements had the strongest effect size in relation to false belief reasoning, with an effect size of 44%, though such studies were less common. Farrar et al. (2017) provide an updated meta-analysis of those studies that contained data on the role of complements and general language as predictors of false belief reasoning. In 10 of the 18 studies (55%) that compared both, the *general language* hypothesis was supported over and above the specific role of complements. These studies used a wide variety of measures to assess “general language ability,” including receptive vocabulary and different measures of syntax development. However, six of these ten negative studies were for Cantonese and Korean. Mental state verbs differ in these Asian languages compared to English, in that the distinction between true and false beliefs is carried lexically in the verb (see Tardif et al., 2007). Nevertheless, the majority of these studies tested complements with communication verbs (except for Cheung, 2006; study 2). Thus Farrar et al. argue that even these cross-linguistic studies on Asian languages can be used to evaluate the relative contribution of complementation versus general language.

Since then, more recent work has used a variety of measures to distinguish the contribution of complements versus more general language, usually taking out the contribution of these skills and then testing whether complements explain added variance. Brandt et al. (2023) and Boeg Thomsen et al. (2021) both contained language measures, and after taking out the contribution of vocabulary and other syntax, found a significant additional contribution of complements measures to false belief understanding in keeping with the *complements facilitate reasoning* hypothesis. An exception mentioned is De Mulder et al. (2019) in a Dutch study, using a complement task that avoided truth contrasts altogether. That task made no contribution to false belief reasoning relative to other language measures such as vocabulary and general syntax. Hence the contribution made by the truth contrasts of non-factive complements is still a live issue (see also de Villiers et al., 2012).

Longitudinal studies are rarer, but they can help identify the direction of influence between the variables, as well as control for the level of initial false belief reasoning. It would be natural to propose that children at three or four years do not yet have the conceptual resources to consider others’ perspectives and mental worlds, leading to errors with false complements as a result of their failures to understand others’ false beliefs. De Villiers and Pyers (2002) studied a small group (*N* = 28) of children over a year in preschool, and tested them at four points on a battery of theory of mind and language tests. Though they had begun the study expecting that false belief understanding might be necessary for comprehending complementation, the reverse turned out to be the case. When children acquired an understanding of sentential complements, then they began to systematically pass false belief tasks. As a control, de Villiers and Pyers took a language sample and looked at the children’s general command of syntax using the IPSYN (Index of Productive Syntax, Scarborough, 1990), an index of the variety and complexity of sentence use. This measure was not as well linked to false belief mastery.

Boeg Thomsen et al. (2021) did a small longitudinal study with 45 English speaking children designed specifically to try to tease apart the potential contribution of two aspects of complement-clause acquisition. As discussed, their complement comprehension task varied from the more common one by not asking complex questions, and by entailing a distractor clause that did not contrast truth. They tested proficiency with the perspective-marking syntactic structure itself, and in a separate task, understanding of the specific mental verbs used, namely *know* versus *think*. As additional language tests, they included memory span, a test of vocabulary, and receptive grammar, and epistemic modals. Furthermore, they avoided complement structures in the false belief task altogether making this one of the most stringent tests to date. They found a robust effect linking third person complements to false belief reasoning when controlling for the rest, as well as an additional effect of mental verb understanding. These studies also are in keeping with the *complements facilitate reasoning* hypothesis.

### 1.4 Executive function and complements

Given two potential explanations of the changes in theory of mind reasoning in the later preschool years, is it possible to decide between the *complements facilitate reasoning hypothesis* and the *executive function* hypothesi*s*? Some have argued that the complement comprehension task (de Villiers and Pyers, 2002) might in itself be an inhibitory control task, in that to answer the critical question, the reality response must be suppressed (Boeg Thomsen et al., 2021). Unfortunately, it is not often that language and executive function are pitted against one another in the same study. In the cross-sectional study by Jenkins and Astington (1996), there was no longer any effect of working memory (a component of EF) in their 3-and 4-year olds’ false belief understanding when controlling for language. Similarly, in Hughes (1998), taking out verbal skills reduced the effects of EF (working memory and inhibitory control) on false belief reasoning in their sample. However, those studies did not consider complements. Low (2010) found that both complement comprehension and executive functioning predicted explicit false belief reasoning in preschoolers, but he could not separate the effects of each of those variables in regression analyses.

Studying children with deafness and delayed language can provide more perspective here. In de Villiers and de Villiers (2012) the oral deaf children were on par with their hearing peers on executive function tasks, but they showed significant delays in false belief tasks even when the language demands of the task were minimized. They report a dissociation of deception and false belief tasks, in that the deaf children were equivalent to their hearing peers on deception games. Furthermore, language, and in particular complement syntax, proved to be the best predictors of false belief reasoning; however, executive function skills, especially inhibitory control, were the best predictors of deception.

A longitudinal study by Farrant et al. (2012) was rich enough to explore the relative contributions of vocabulary, general language, executive functioning and complements to false belief reasoning in English-speaking preschool children. Farrant et al. added to the model the variable of mind-mindedness (Meins et al., 2002), predicting that variation in maternal input about mental states (via perhaps the *discourse content* hypothesis) would predict children’s ability on sentence complements, which would then predict false belief understanding. Their sample included 91 typically-developing Australian children studied twice across a year. Importantly, the effects of variation in maternal mental talk were completely mediated by the children’s own competence at sentential complements, which predicted their belief ability. Cognitive flexibility was a further predictor, and the direction of effect was that sentential complements predicted this executive function index rather than vice versa. However, the Farrant study had a relatively small sample size for the number of variables, and they did not use structural equation modeling for the longitudinal portion of their study. The recent study by Boeg Thomsen et al. (2021) also tested the effects of inhibitory control as well as several other language variables in their longitudinal work, but there is a limit on the power of linear regressions with so few participants (45) and so many test variables. The regressions revealed a bidirectional effect of complements and false belief reasoning across time.

In sum, the existing studies are typically too small and contain too many variables for a sufficiently powerful statistical technique such as structural equation modeling that can differentiate direction of effects in a longitudinal study.

### 1.5 The current study

In the current study we had the opportunity to test the different theoretical models of false belief development on a large sample of low-income children studied over the course of several years on a research grant attached to a preschool curricular intervention study (Lonigan et al., 2015). In our project the children received a large battery of language, executive function, and theory of mind measures, and these were repeated several times over the course of the study, making this an ideal group to test competing models. Lonigan et al. (2015) is a report on the curriculum intervention, using some measures that overlapped with the current study but it does not include the measures of complex language, executive function or theory of mind used here.

The intervention study itself was a large-scale cluster-randomized investigation of the effects of an integrated literacy-and math-focused preschool curriculum. The basic curriculum incorporated central elements of the Literacy Express Curriculum (Lonigan et al., 2005) and Pre-K Mathematics (Klein et al., 2002), two preschool curricula rated as effective by the US Department of Education’s What Works Clearinghouse. One hundred and ten center-based preschools serving low-income communities in the Houston, TX and Tallahassee, FL areas were randomly assigned to one of three conditions: 1. the base curriculum with added explicit socio-emotional instruction [the Promoting Alternative Strategies Thinking curriculum (PATHS) (Domitrovich et al., 2007)]; 2. the base literacy and math curriculum and general classroom and behavior management instruction for the teachers, but with no explicit lessons and activities targeting socio-emotional skills (called the *Implicit* Socio-Emotional Condition); or 3. a “business-as-usual” control condition. There were significant positive impacts of the two versions of the curriculum on language, phonological awareness, math, and socioemotional outcomes. There were no added benefits to academic or socioemotional outcomes for the children receiving *explicit* socioemotional instruction (Lonigan et al., 2015). For this reason, in the current research we combine the first two conditions into the Intervention Condition and compare that to the Control Condition to explore the effects of the intervention on false belief reasoning in our sample of children.

Our study is unique in using a longitudinal design with a very large N (258 preschoolers), allowing for powerful statistical analyses to separate the effects of different possible predictors of the development of false belief reasoning. Both the familiar verbal false belief reasoning tasks (unseen object displacement and unexpected contents) and low verbal tasks (thought-bubble picture narratives) were employed, with enough false belief questions (14) to provide considerable variance in the dependent variable to tease apart effects of different independent variables. Most importantly, longitudinal measures of inhibitory control and several relevant measures of language acquisition were taken. The language measures included expressive vocabulary, morphosyntax, and complement comprehension and there were enough items in each measure to provide sufficient variance to separate the effects, if any, of each aspect of language. Prior studies of the effects of children’s language acquisition on their false belief reasoning have often had too few subjects for reliable statistical analyses, have taken too few measures of false belief reasoning, have not pitted executive functioning against language, or have not separated the effects of different aspects of language.

### 1.6 Predictions

Based on our review of past work, we make the following predictions:

1. Curricular intervention that enriches socio-emotional understanding is expected to improve false belief reasoning.
2. Inhibitory control will predict false belief reasoning concurrently but not across time when other variables such as language are controlled.
3. Language measures will predict false belief understanding both concurrently and over time.
4. Complement comprehension will be a significant predictor of false belief reasoning over and above other control variables, including more general language measures
5. Complement comprehension will be correlated with inhibitory control, but complements will be a more important proximal predictor of false belief reasoning compared to inhibitory control.

## 2 Method

### 2.1 Participants

The participants were 258 children from subsidized^1^ preschools in Texas and Florida. 90.4% of the children came from low-income families and were eligible for free lunches. Tables 1 and 2 give the sample statistics on gender and ethnicity. They were recruited as a part of a curriculum intervention project funded by the National Institutes of Health, the School Readiness Research Consortium. All of the children were assessed twice on a battery of socio-emotional, cognitive, quantitative, language and pre-reading tasks during the preschool year. The first time was in September at the beginning of the school year before the curriculum intervention (Time 1) and the second time was in April/May at the end of the school year, after the intervention (Time 3). The children ranged from 3.3 to 5.3 years old (Mean = 4.58, sd. = 0.34) at Time 1 and from 3.8 to 5.9 years old (Mean = 5.11, sd. = 0.34) at Time 3.

**Table 1:** Sample by ethnicity.

Ethnicity	*N* (%)
African American	141 (54.7)
White	78 (30.2)
Hispanic	26 (10.1)
Other	13 (5.0)

Out of 760 preschoolers who completed Time 3 of the NIH curriculum project, the 258 children in the present study included only those children who completed all of the verbal and low verbal false belief tasks as well as all of the relevant tests of inhibitory control and language acquisition at both Time 1 and Time 3. We thus avoided the issue of imputation of missing data. Given the intensity of the testing schedule across four projects, some loss of data was inevitable, especially as these were low-income day care centers with considerable changeover and parents who moved a lot. However, there is no evidence that the children that we excluded for missing tests (170) constituted a different population than those who completed all the measures (258). We checked this by comparing the two groups on the vocabulary measure, which was present for 100% of the sample, and also age. The groups did not differ statistically on either measure. Children who were not monolingual speakers of English were also excluded. Parents had to return a questionnaire on home language use to determine if the child was monolingual in English (183 of these were missing). Children who were reported by their primary caregiver as having more than 10% Spanish input and/or use were excluded from the present study to eliminate possible effects of bilingualism. There were 149 such exclusions.

**Table 2:** Gender make-up of sample.

Gender	*N* (%)
Male	126 (48.8)
Female	132 (51.2)

### 2.2 Procedure

The children were tested individually on the full assessment battery in three one-hour testing sessions. The testing took place over several days, contingent on the availability of particular children in the center on a given day, and because the overall test battery including this study was very large. Tests were given in a fixed order across the children in each of these sessions.

#### 2.2.1 Measures for the present study

##### Background measures

###### Nonverbal IQ

Children’s nonverbal IQ was assessed using the Pattern Analysis subtest of the Stanford-Binet IQ test, which measured children’s capacity to recognize abstract visual patterns and to solve pattern matching problems. This test was carried out at Time 2, midway between Time 1 and Time 3.

###### Verbal memory

Verbal memory was assessed with the Word Span subtest on the Comprehensive Test of Phonological Processing (CTOPP) (Wagner et al., 1999). The children were asked to repeat a string of familiar English words said quickly by the tester (e.g., “fish, bed, dog”) in the same order as they had been produced. The strings of words increased in length until the child failed to repeat them accurately. This test was also administered at Time 2.

##### False belief reasoning

###### Verbal FB reasoning tasks

####### Unexpected contents

The unexpected contents task is a standard task about whether children can remember their earlier false belief about the expected contents of a familiar container after knowing the true content, and whether they can predict others’ false belief about the container’s contents (Perner et al., 1987). Each participant was shown a box (e.g., a crayon box) and the tester asked them what they believed to be in the box. After the child’s answer, the tester opened the box and showed that it actually contained an unexpected item (e.g., a spoon). The child was then asked what they thought was inside the box before it was opened, and what their friend or a toy character would think before it was opened. Each child was tested on two different containers at T1 and another two containers at T3, so they could not just remember what was in the container from T1 to T3. Our prior research over some twenty years had shown the four containers with unexpected contents to produce essentially equivalent responses from preschoolers.

####### Unseen object displacement

The unseen object displacement task is a modification of the Sally-Anne test (Wimmer and Perner, 1983), which is the standard task measuring whether the children can predict a character’s behavior after successfully reasoning about their false belief (de Villiers and de Villiers, 2012). Children were told two different picture-supported stories, each involving two characters. One character (e.g., a boy) put an object (e.g., a basketball) at one place and left the room. The other character, without deceptive intent, put the object somewhere else. The children were then asked where the boy would first look for the basketball when he came back into the room and why he would look there for the ball. Different stories were used at T1 and T3. Prior research had shown that the four stories produced very similar responses from preschoolers.

Children’s performance received a score of either 1 (for a correct response) or 0 (for an incorrect response) for each verbal false belief question across the Unexpected Contents and Unseen Displacement tasks. This produced a total verbal false belief score out of 8 for each participant.

###### Low-verbal FB tasks

####### Thought bubbles tasks

Both 2-picture sequences (Woolfe et al., 2002) and 4-picture sequences (de Villiers and de Villiers, 2012) were employed. The low-verbal tasks were tested together. Three training items were completed before the administration of low-verbal tasks to make sure the children understood the nature of thought-bubbles as representing the contents of what a character was thinking. Children who failed the training would not be given the low-verbal tasks.

The 2-picture sequence procedure (Woolfe et al., 2002) contained three false belief trials and two true belief trial. The children were shown pictures in which a character was thinking something but the thought bubble was empty, and were asked to choose from three pictured objects what they thought should go into the bubble. An example of a false belief trial showed a picture of a boy fishing with a bending rod. The end of the line and the hook were covered by a flap. The tester instructed the child to lift the flap and a boot was revealed to the child but not the character in the picture. Then the tester pointed to the thought bubble on the boy’s head and asked “What goes it here?.” The child would then choose among three pictures: a fish (false belief), a boot (reality) and a bird (distraction). An example of a true belief trial would be identical to the false belief trials except it would reveal a fish rather than a boot.

The 4-picture-sequence procedure (de Villiers and de Villiers, 2012) included three false belief trials and one true belief trial. In these picture sequences, one of the two characters replaced the contents of a familiar container (e.g., playdough) with an unusual object (e.g., worms). A second character either observed or did not observe this replacement. The children were then asked what should be put into the thought bubble above the second character’s head before s/he opened the container (e.g., a picture of playdough or a picture of worms). The picture was drawn in a way such that it was reasonable for the character to want to find either object that could be in the container.

For each false belief trial, the child received a score of 1 (for a correct answer) or 0 (for an incorrect answer). The total score out of 6 false belief trials was then recorded as the child’s low-verbal false belief score.

##### Language

###### Vocabulary

Children’s achieved vocabulary in English was assessed with the Expressive One Word Picture Vocabulary Test (EOWPVT-R; Brownell, 2000) at both T1 and T3. They were asked to name the objects, actions, or concepts shown in colored pictures. In all our analyses we used the children’s raw scores on the test, the number of words correctly produced.

###### Morphosyntax

As an additional measure of the children’s general language development, we used their raw scores on the morphosyntax items of the Risk Subtest of the Diagnostic Evaluation of Language Variation – Screening Test (DELV-ST; Seymour et al., 2003). This test was used because it assesses African-American children’s acquisition of morphosyntax that is neutral with respect to differences between African American English (AAE) and Mainstream American English (MAE). We did not wish to use standardized tests of MAE that are biased against children speaking other dialects of English, since a high proportion of the children in our study were African American (See Table 1), many of them AAE speakers.

###### False complement comprehension

Children’s ability to process syntactic structures that differentiate what a person said from reality was tested using the task developed by de Villiers and Pyers (2002). The False Complement Comprehension task contains 8 trials testing whether the children can hold the complement structure associated with the verb “say” in memory and successfully reproduce it. In each trial, a story was told along with pictures about one character saying something which was different from reality. For example, “The woman said there was a bug in her cereal. But look, it was just a raisin!” At the end of the story the tester asked “What did the woman say was in her cereal?” For each trial, the child received a score of either 1 (passed) or 0 (failed). The total score out of 8 was then recorded.

##### Inhibitory control

###### Bird and dragon task

The Bird and Dragon Task is a simplified version of “Simon Says” (Reed et al., 1984; Kochanska et al., 1996). In this task children need to selectively inhibit commanded actions. In 10 familiarization trials, the tester first asked the children to imitate self-directed action (e.g., “Touch your ears”). Then the tester introduced two puppets: a “nice bird” and a “naughty dragon.” The instruction was to follow the bird’s commands but not the dragon’s. Children’s performance on each dragon command received a score of 1 (performed a full movement), 2 (performed a wrong movement), 3 (performed a partial movement), or 4 (did not move), and the mean of scores on 7 dragon commands was recorded as the “inhibition score.”

###### Knock-tap task

The Knock-Tap Task required children to be able to switch from imitating hand actions to doing the opposite action (Korkman et al., 1998). First the children were asked to imitate the examiner by either knocking with a closed fist or tapping with an open palm on a box for 8 trials. Then for 8 pseudorandom opposite trials the children had to tap when the examiner knocked and knock when the examiner tapped. Thus, in this task the children had to inhibit the prepotent response of imitating the tester’s hand action, the response that had just been primed. Percentage of correct responses over 8 opposite trials was recorded for each child.

## 3 Results

### 3.1 Background measures

Our analysis of the factors contributing to false belief reasoning in the children began with two regression analyses. The first looks at the cross-sectional predictors at T3, taking out the level of FB at T1. The second takes advantage of the longitudinal data to look also at predictors from T1 to T3. Finally, we construct a structural equation model to look at the interactive effect of the predictors. But first an outcome score, a composite of false belief measures was required. Children’s scores on the four false belief tasks were significantly intercorrelated (see Table 3), so a composite Total FB score was created by adding together the responses to all of the FB questions. Scores varied from 0 to 14 correct out of 14.

**Table 3:** Intercorrelations between the different False Belief tasks at Time 3 (*N* = 258).

	Unseen	Unexpected	Thought Bubble 2-Picture
	Displacement	Contents	
Unexpected contents	0.316***		
Thought bubble 2-Picture	0.307***	0.296***	
Thought bubble 4-Picture	0.360***	0.312***	0.380***

Although we intended to create a composite executive function score, a composite inhibitory control score was not calculated from the Bear-Dragon and Knock-Tap Test because the partial correlations between performances on the two measures were low (*r* = 0.133, *p* = 0.033 at T1; *r* = 0.114, *p* = 0.069 at T3; partial correlations controlling for Age). The Bear-Dragon test was selected as the predictor because it had the strongest bivariate correlation with False Belief.

The children showed significant longitudinal growth over the 6 months between T1 and T3 in their false belief reasoning, in each of the Inhibitory Control tasks, and in the three Language measures (see Table 4).

**Table 4:** Longitudinal growth in false belief reasoning, inhibitory control, and language measures between Time 1 (early in the preschool year) and Time 3 (late in the preschool year).

Measure	Time 1	Time 3	*t*	df	*p*
ToM					
False Belief	5.90 (3.24)	8.98 (3.02)	17.50	258	0.000***
Inhibitory control					
Bear-Dragon	0.74 (0.35)	0.93 (0.18)	8.49	258	0.001**
Knock-Tap	0.68 (0.34)	0.82 (0.26)	5.64	258	0.002**
Language					
Morphosyntax (DELV-ST)	5.63 (1.12)	6.27 (0.085)	11.07	258	0.000***
Vocabulary (EOWPVT-R)	38.75 (11.23)	46.60 (12.09)	15.11	258	0.000***
Complements	6.02 (2.05)	7.34 (1.34)	11.63	258	0.000***

There were significant effects of both Gender and Curriculum Intervention on the children’s total FB score at T3 (Gender: F (N = 132) FB mean = 9.53/14, M (N = 126) FB mean = 8.40/14; *t*(256) = 3.03, *p* = 0.003) (Intervention: INT (N = 215) FB mean = 9.25/14, CONTROL (N = 43) FB mean = 7.63/14; *t*(256) = 3.27, *p* = 0.001). Therefore, both Gender and Intervention were entered as background predictor variables in the subsequent analyses of the relationship between inhibitory control and language development and the children’s FB reasoning toward the end of preschool. Predictors of Total FB reasoning scores at T3 were examined using both linear hierarchical multiple regressions and structural equation modeling.

### 3.2 Cross-sectional predictors of false belief reasoning at time 3: regression 1

First, a cross-sectional multiple regression was performed relating T3 raw scores on the Bear-Dragon Game and the three Language measures to the children’s T3 Total FB scores. The Language measures were kept separate to investigate the independent effects of Morphosyntax, Vocabulary, and Complements on FB reasoning, although the regression also showed the combined effects of the three on the variance in total FB score at T3. Predictors were entered in four blocks: first, Background Variables [Age at T3, Gender (1 = Female, 0 = Male), and Intervention (1 = Intervention Group, 0 = Control Group)]; second, the children’s T1 FB scores; third, performance on the Bear-Dragon task; and finally, the three Language measures (see Table 5).

**Table 5:** Cross-sectional hierarchical linear regression predicting T3 False Belief from Background variables (age, gender and intervention), T1 false belief scores, inhibitory control, and language measures.

	∆R^2^	*F*(df)	*p*	Predictor	ß	*t*	*p*
Background	0.157	15.80 (3,254)	0.000***	T3 Age	0.29	5.03	0.000***
				Gender	0.188	3.27	0.001**
				Intervention	0.184	3.2	0.002**
T1 ToM	0.247	42.96 (1,253)	0.000***	T1 False Belief	0.536	10.25	0.000***
Inhibitory control	0.009	3.85 (1,252)	0.050*	T3 Bear-Dragon	0.097	1.96	0.050*
Language	0.127	22.94 (3,249)	0.000***	T3 Morphosyntax	−0.049	−0.92	ns
				T3 Vocabulary	0.204	3.86	0.000***
				T3 Complements	0.306	6.06	0.000***

The Background variables accounted for a significant proportion of the variance in T3 FB scores (R^2^ = 0.157, *p* = 0.000) and each of the three variables was a significant contributor (see Table 5). Children’s FB scores at T1 were major predictors of their FB reasoning at T3, accounting for an additional 24.7% of the variance (*p* < 0.001). Performance on the Bear-Dragon Game at T3 was also a significant predictor of later FB reasoning (∆R^2^ = 0.009, *p* = 0.050), although the additional variance accounted for by this measure was small. Finally, the T3 Language measures added substantially to the prediction of T3 Total FB scores (∆R^2^ = 0.127, *p* = 0.000). Of the Language measures, both Expressive Vocabulary and Complement Comprehension were significant independent predictors of T3 FB (Vocabulary: *B* = 0.204, *t* = 3.86, *p* < 0.001; Complements: *B* = 0.306, *t* = 6.60, *p* < 0.001), but Morphosyntax was not in itself a significant predictor.

### 3.3 Longitudinal predictors of false belief reasoning at time 3: regression 2

The second regression considered the possible longitudinal effects of the additional measures taken at T1, not just the continuity in FB scores from T1 to T3. For the longitudinal regression analysis between T1 and T3, the predictor variables were also entered in four blocks (Table 6). First, the three background variables were entered: Age at T3, Gender, and Intervention. Next, the children’s T1 FB scores were entered. Then scores on the Bear-Dragon Inhibitory Control game at T1 were entered. Finally, the three Language measures from T1 were entered as a block: DELV-ST dialect neutral Morphosyntax, Expressive Vocabulary, and Complement Comprehension. Thus, the first two blocks of this regression analysis were the same as the cross-sectional T3 analysis, but longitudinal Inhibitory Control and Language predictors were entered into this regression.

**Table 6:** Longitudinal hierarchical linear regression predicting T3 false belief from background variables (age, gender and intervention), T1 false belief scores, inhibitory control, and language measures.

	∆R^2^	*F*(df)	*p*	Predictor	ß	*t*	*p*
Background	0.157	15.80 (3,254)	0.000***	T3 Age	0.29	5.03	0.000***
				Gender	0.188	3.27	0.001**
				Intervention	0.184	3.2	0.002**
T1 FB	0.247	42.96 (1,253)	0.000***	T1 False Belief	0.536	10.25	0.000***
Inhibitory Control	0.03	13.39 (1,252)	0.000***	T1 Bear-Dragon	0.188	3.66	0.000***
Language	0.071	11.93 (3,249)	0.000***	T1 Morphosyntax	0.004	0.07	ns
				T1 Vocabulary	0.261	4.65	0.000***
				T1 Complements	0.138	2.55	0.011*

*p < 0.05, **p < 0.01, ***p < 0.001.

The results for Block 3 and Block 4 of the longitudinal regression were very similar to those from the cross-sectional analysis: performance on the T1 Bear-Dragon game added significantly to the variance in T3 FB scores accounted for by the variables in Blocks 1 and 2 (∆R^2^ = 0.030, *p* = 0.000) and Expressive Vocabulary and Complement Comprehension at T1 were each independent predictors of later T3 FB reasoning (Vocabulary: B = 0.261, *t* = 4.65,*p* < 0.001; Complements: B = 0.138, *t* = 2.55, *p* < 0.011). The Language measures as a group accounted for 7.1% of the variance in FB scores (*p* < 0.0001). Morphosyntax scores on the DELV-ST did not add any predictiveness to the Language measures.

### 3.4 Structural equation model

**Figure 1:**
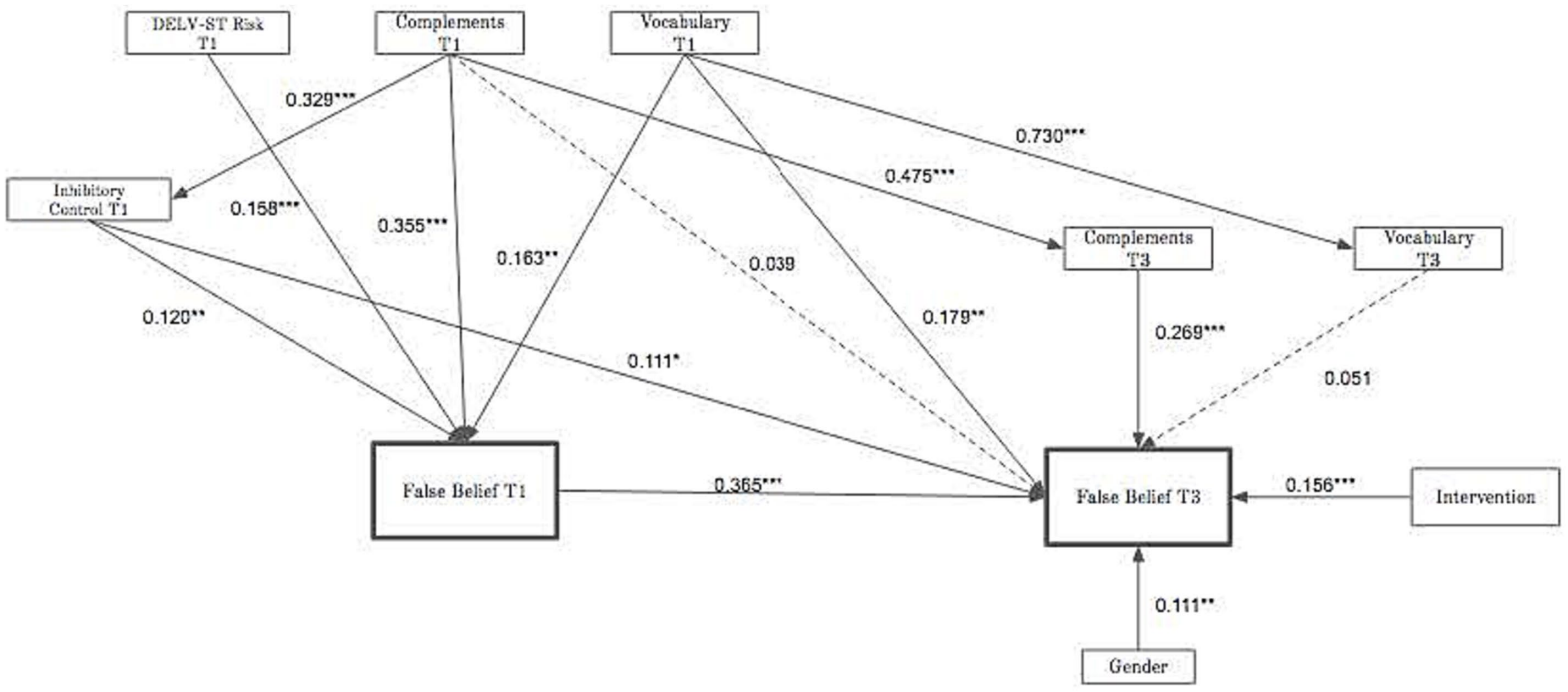
Concurrent and longitudinal structural equation model of the relationships between inhibitory control (Bear-Dragon game), the three language measures, and total false belief scores at Tl and T3. Effects of gender and curriculum intervention are also part of the model. Raw scores were entered for the Bear-Dragon game, DELV-ST Morphosyntax, complement comprehension, and vocabulary at Tl and T3 . The figure gives standardized parameter estimates for the hypothesized longitudinal SEM. All reported estimates are the maximum likelihood standardized point estimates. χ^2^ (21, *N* = 258) = 38.008, *p* = 0.013; Comparative Fit Index = 0.978; Tucker-Lewis Index = 0.954; Root Mean Square Error of Approximation (RMSEA) = 0.056; Standardized Root Mean Square Residual (SRMSR) = 0.068. All error covariances were calculated but are not shown.

The power of structural equation methods lies in their ability to model complex interactions and direction of effects, especially in longitudinal data. A structural equation model was fitted to the data from T1 and T3 using the same predictor variables and outcome measures as the regressions (see Figure 1). The model shows both concurrent effects of Inhibitory Control and Language measures at T1 and T3 on FB scores at T1 and T3, and longitudinal effects of the T1 measures on T3 scores. The final model is an excellent fit to the data, with fit indices >0.95 and RMSEA and SRMSR values <0.07.

The SEM complements the findings of the two regression analyses. Inhibitory Control (Bear-Dragon) at T1 has significant direct effects on FB at T1 (Standardized Parameter (SP) = 0.12, *p* < 0.001) and longitudinally at T3 (SP = 0.111, *p* < 0.05). Bear-Dragon scores at T3 did not add to the fit of the model and standardized parameter estimates between that measure and FB at T3 were not statistically significant, so that variable was excluded from the final model.

Of the Language measures, Vocabulary and Complement Comprehension have stronger effects than Morphosyntax. Morphosyntax scores on the DELV-ST at T1 are significantly related to FB at T1 (SP = 0.158, *p* < 0.001), but not to FB at T3. T3 Morphosyntax scores did not have significant effects on T3 FB, so they are excluded from the model.

Complement Comprehension scores have direct effects on FB at T1 (SP = 0.355, *p* < 0.001), but not longitudinally on FB at T3. Instead, they have strong indirect effects on T3 FB through their effects on T3 Complement Comprehension (SP = 0.475, *p* < 0.001). T3 Complement Comprehension is significantly related to FB at T3 (SP = 0.269, *p* < 0.001).

Expressive Vocabulary at T1 has significant direct effects on both T1 (SP = 0.163, *p* < 0.001) and T3 FB (SP = 0.179, *p* < 0.001), and T1 Vocabulary has a significant longitudinal effect on T3 Vocabulary (SP = 0.730, *p* < 0.001); but T3 Vocabulary is not significantly related to T3 FB in this model. The best fit directional pathway between Inhibitory Control at T1 and Complement Comprehension at T1 goes from Complements to Inhibitory Control (SP = 0.329, *p* < 0.001).

## 4 Discussion

The data analyses allow separation of the various influences on false belief reasoning over time, taking into account the child’s initial level of success. Of course, these results do not rule out the possibility that different developmental paths might occur in other cultures and other languages, which is why a broader net of research must still be cast. This is the first study of a large sample of poorly-resourced children in the US, the majority of whom were African American or Hispanic.

Our first prediction concerned the effects of the intervention on false belief reasoning. Since the interventions were randomized, all the groups contained an equivalent mix of children from low-income households with parents of low educational achievement. The larger group of researchers had an interest in curricular improvements that could be implemented in low-income day centers that could pay off in early schooling, and as such, were not focused on either syntax nor theory of mind (Lonigan et al., 2015). However, the intensive exposure to books, exercises and materials that focused on emotion regulation undoubtedly enriched the children’s exposure to language about the mind, and it paid off in the strong effects of the intervention versus business-as-usual uncovered in the analyses. These results cannot distinguish between the various hypotheses, but are at least compatible with the *discourse content* hypothesis, namely, that the children’s development of theory of mind is enriched by more mind-talk (Cutting and Dunn, 1999; Meins et al., 2002). They reinforce and refine the Lonigan et al. (2015) results in that they demonstrate an effect of teacher-delivered, richer day care curricula on theory of mind development in the least-resourced children.

The finding that the intervention impacted theory of mind outcomes for the children was not previously reported, as the published report from the longitudinal study looked only at emotion understanding and parental reports of social development (Lonigan et al., 2015). Including all the groups may have increased the variance in achievements of the sample. The regression already accounted for the variance due to intervention, age and gender, and the language variables then entered were additional sources of influence.

Like some other studies, we also found an effect of gender on false belief reasoning, a fact that is not fully explained. Some prior studies with preschool-aged children have found girls to show slightly better performance on emotion understanding and false belief tasks (Banerjee, 1997; Charman and Clements, 2002), and some found differences in later theory of mind skills (Calero et al., 2013; Kuhnert et al., 2017). However, several others have found no difference (Devine and Hughes, 2012; Mathieson and Banerjee, 2011; Walker, 2005). A gender effect has sometimes been attributed to the differential discourse of parents by the gender of their child, with girls receiving a higher volume of talk about people and relationships, hence mental states (Charman and Clements, 2002). The other possibility is that it reflects the finding that girls outpace boys in language development, though that difference has usually been found to be limited to the early years. In the current data, the girls showed small but statistically significant differences in each of the language skills relative to the boys.

Turning to the theoretical significance of the findings, one can see reflections here of all the major theoretical proposals of factors that influence development. The results of the SEM turned out to be more subtle than our initial predictions. First, it is evident that the child’s *executive functioning*, indexed here by inhibitory control, exerts differential influence across ages. Whether measured at T1 or at T3, inhibitory control has a small but significant effect on false belief reasoning at T3 as predicted. However, the SEM model makes clear that the variance in inhibitory control at T1 has its significant influence by affecting false beliefs at T1. By T3, inhibitory control makes no difference, primarily because the variance at T3 is so limited: the majority get full points on the measure. Recall also that our measure was limited to a single task, because of the lack of correlation between the two inhibitory control tasks with which we began. Surprisingly, inhibitory control was not a contributor to complement comprehension: the SEM makes clear that the direction of influence is the reverse. The complement comprehension test has been seen as potentially a kind of inhibitory control task, but that is not evident here. Farrant et al. (2012) found the same direction of effect in their SEM model with typical children, namely, that sentential complements predicted executive function rather than vice versa.

A picture is beginning to emerge of how the different factors contribute to the two processes involved in explicit false belief reasoning. Inhibitory control would seem to be needed for the second step, of suppressing the prepotent response. If the representation of alternatives is appropriate then inhibitory control can assist, but if the representation is inadequate then inhibitory control will not help. The representation must be built first, and language, specifically complementation, is the ideal structure. A child could have developed inhibitory control for other decisions but still fail on both complements and on false beliefs, as in the case of the oral deaf children in de Villiers and de Villiers (2012). A child could theoretically have complements but not inhibitory control, perhaps a child with ADHD, though this has not been explored. The interpretation is partly supported by the results of a recent training study (Boeg Thomsen et al., 2024) that demonstrates that preschool children benefited most from complement training when they had better executive function skills to begin with.

Second, there is support here for the *general language* hypothesis such as vocabulary development or general morphosyntax, as predicted. The child’s vocabulary at T1 matters for false belief understanding both at T1 and at T3, but like inhibitory control, the effect of T3 vocabulary on false belief understanding at T3 disappears, and a similar pattern occurs with morphosyntax, despite there being plenty of variance in both still at T3. A major effect at both time points is complement comprehension. What matters at T3 is complement comprehension at T3. That is, the proximal effect by the final stage is the children’s complement comprehension measured then. The other influences at an earlier point affect the earlier stages of false belief understanding comprehension. The average false belief understanding score at T1 was 5.46, well below chance for 14 items (*p* = 0.001), but by T3, it was 10.52 (above chance, *p* = 0.001) with a distribution that shows more equal proportions of passers and failers. It is therefore possible that vocabulary, morphosyntax and inhibitory control each make a difference in getting any points at all on the false belief task, but once that is underway, it is complement comprehension that matters. Of course, the present study is still limited by the range of language measures used, and it remains possible that other complex language could play a role in enhancing false belief reasoning (Schroeder et al., 2021; Charnavel et al., 2024).

The importance of these findings is that they reveal how different studies, with small and slightly different age samples, can come up with different results in which executive function, vocabulary, or complement syntax might make different contributions statistically. Furthermore, enriched talk about the mind via specially prepared curricula or books may also be effective in promoting growth. Only with longitudinal data of this degree of richness can a full developmental story emerge. However, though the age span covered here is quite broad (3.3 to 5.9), the longitudinal time interval was still relatively short as the children averaged 4.6 at T1 and 5.1 at T3.

The result that complements predict false belief reasoning confirms the predictions and is compatible with the data from a number of training studies that have set out to test whether false complements can be trained, and if so, whether that linguistic training impacts false belief reasoning. An excellent review of existing training studies is provided in Boeg Thomsen et al. (2024). Though some studies do not conclusively demonstrate a specific effect of complementation (Hale and Tager-Flusberg, 2003; Lohmann and Tomasello, 2003), others provide convincing evidence that specific training on complements has positive effects on later false belief reasoning (Mo et al., 2014; Durrleman et al., 2019; Durrleman and de Villiers, 2024; Boeg Thomsen et al., 2024). Training studies, since they constitute intervention in the causal chain, can be considered the ultimate experimental test of the causal hypothesis, unlike even the best of longitudinal studies that do not train. But training studies do not settle the question of whether this is the normal path of development. Together with reliable longitudinal data, the case is more robust.

Several theorists have maintained that language may be prerequisite for, or an important determinant of, explicit but not implicit theory of mind (Apperly and Butterfill, 2009; Low, 2010). Low and Watts (2013) suggested a signature limit (Apperly and Butterfill, 2009) on the implicit theory of mind system. The proposal is that the implicit system may be capable of tracking only a subset of mental states, such as a protagonist’s beliefs about the location of an object, but not beliefs about the object’s properties or identity. Results of a study by Fizke et al. (2017) support this proposal. Perhaps language is more influential, or necessary, for beliefs about object identity. In unseen displacement the child must anticipate where a person will look, and that could be done by a system that recognizes another’s goals or intentions, but not necessarily beliefs. However, object identity tasks require representing how the other person conceives of an object, and that is a more demanding representation (Rakoczy, 2017). More precise work is needed to test this distinction.

The overall findings of the study are compatible with the claim that sentential complements play a significant role in explicit false belief reasoning development, separate from general morphosyntax, vocabulary and executive function. Many questions remain about the scope of the finding cross linguistically, as so few languages have so far been investigated and the range of mental state expressions is diverse, as are the techniques for assessing them. Questions also remain about the role of implicit theory of mind as an additional component of influence on the variance in children’s achievement. More research is needed on whether there is real continuity of skills across the whole span from infancy to higher order belief at age six or seven, and what effects there might be of the type of belief engaged in the task.

## Data Availability

The raw data supporting the conclusions of this article will be made available by the authors, without undue reservation.

## Footnotes

^1^In the US, Early Education and Care (EEC) provides subsidies to families in need to help cover the cost of child care. Child care programs accepting these subsidies use EEC’s Child Care Financial Assistance (CCFA) system for documenting family eligibility, tracking child attendance, and submitting requests for reimbursement.
